# Population-level coordination of pigment response in individual cyanobacterial cells under altered nitrogen levels

**DOI:** 10.1007/s11120-017-0422-7

**Published:** 2017-07-21

**Authors:** Jaclyn Murton, Aparna Nagarajan, Amelia Y. Nguyen, Michelle Liberton, Harmony A. Hancock, Himadri B. Pakrasi, Jerilyn A. Timlin

**Affiliations:** 1 Bioenergy and Defense Technologies, Sandia National Laboratories, Albuquerque, NM 87123 USA; 2 Department of Biology, Washington University, St. Louis, MO 63130 USA; 3 Present Address: Renewable and Sustainable Energy Institute, University of Colorado Boulder, Boulder, CO 80309 USA; 4 Present Address: United States Environmental Protection Agency, Washington, DC 20460 USA; 5 Present Address: Department of Biological Sciences, Old Dominion University, Norfolk, VA 23529 USA

**Keywords:** Cyanobacteria, Photosynthesis, Phycobilisome, Nitrogen, Hyperspectral confocal fluorescence microscopy, Multivariate analysis

## Abstract

Cyanobacterial phycobilisome (PBS) pigment-protein complexes harvest light and transfer the energy to reaction centers. Previous ensemble studies have shown that cyanobacteria respond to changes in nutrient availability by modifying the structure of PBS complexes, but this process has not been visualized for individual pigments at the single-cell level due to spectral overlap. We characterized the response of four key photosynthetic pigments to nitrogen depletion and repletion at the subcellular level in individual, live *Synechocystis* sp. PCC 6803 cells using hyperspectral confocal fluorescence microscopy and multivariate image analysis. Our results revealed that PBS degradation and re-synthesis comprise a rapid response to nitrogen fluctuations, with coordinated populations of cells undergoing pigment modifications. Chlorophyll fluorescence originating from photosystem I and II decreased during nitrogen starvation, but no alteration in subcellular chlorophyll localization was found. We observed differential rod and core pigment responses to nitrogen deprivation, suggesting that PBS complexes undergo a stepwise degradation process.

**Electronic supplementary material:**

The online version of this article (doi:10.1007/s11120-017-0422-7) contains supplementary material, which is available to authorized users.

## Introduction

Cyanobacteria are unicellular photosynthetic microbes that played a central role in oxygenating the Earth’s early atmosphere (Buick 2008). Cyanobacteria are found ubiquitously throughout the biosphere and are crucial contributors to the global carbon and nitrogen cycles (Schwarz 2005; Wegener et al. 2010). These organisms have varied morphologies, including spherical, rod-shaped, and filamentous forms, and have the metabolic flexibility to thrive in diverse environments throughout terrestrial, freshwater, and marine habitats. The ability of cyanobacteria to use light energy to fix carbon dioxide and produce oxygen makes these organisms of critical ecological importance and of particular interest in biotechnological studies.

Cyanobacteria contain pigment-proteins that function to harvest and transfer light energy to the reaction centers in the thylakoid membranes that power photochemistry. The blue bilin and green chlorophyll (Chl) pigments impart the characteristic blue-green color to cyanobacteria. The bilin-containing phycobilisome (PBS) complexes are the main light-harvesting antenna in cyanobacteria (Adir et al. 2006). Bilins are covalently bound to proteins to form phycobiliproteins, which are of three major types: phycocyanin (PC), allophycocyanin (APC), and phycoerythrin (PE). These phycobiliproteins serve as the building blocks of the PBS pigment-protein complexes, which are giant membrane extrinsic structures of 3–5 MDa in size (Adir et al. 2006). In a well-studied cyanobacterium, *Synechocystis sp*. PCC 6803 (hereafter referred to as *Synechocystis* 6803), PBS complexes are hemidiscoidal structures consisting of a tricylindrical APC central core and six PC peripheral rods radiating from the core (MacColl 1998). The pigmented phycobiliproteins are interspaced with non-pigmented linker proteins in the PBS structure (Watanabe and Ikeuchi 2013). PBS may account for up to 60% of the total soluble protein in the cell, thus serving as a large cellular nitrogen reserve (Bogorad 1975).

Changes in light conditions and nutrient availability can lead to modifications in pigment composition, abundance, and location aimed to optimize light harvesting and energy production (Grossman et al. 2001; Wegener et al. 2010). The dynamic nature of the PBS can be observed during nutrient deprivation (e.g., nitrogen, sulfur, or phosphorus depletion), when cells bleach due to the degradation of PBS (Collier and Grossman 1992). PBS degradation likely supplies macronutrients for cellular use during nutrient deprivation conditions and may prevent photosystems from undergoing photoinhibition and production of harmful radical species (Adir et al. 2006). Consequently, PBS degradation plays an important role in cell survival.

Several proteins involved in PBS degradation have been identified. An essential factor for the ATP-dependent degradation of PBS is non-bleaching A (NblA) (Collier and Grossman 1994). NblA triggers the degradation of PBS in cyanobacteria by serving as an adapter protein to facilitate the interaction of a protease with phycobiliproteins (Baier et al. 2014, 2001; Karradt et al. 2008; Nguyen et al. 2017). Additional proteins including NblR, NblS, NblB, RpaB, and NtcA have been identified that function with NblA to regulate PBS degradation during nutrient deprivation (Dolganov and Grossman 1999; Grossman et al. 1993; van Waasbergen et al. 2002). Physiological experiments conducted during nutrient starvation have shown that essentially all PBS complexes are degraded in wild-type cells within 48 h (Collier and Grossman 1994; Li and Sherman 2002). Following long-term nitrogen deprivation (e.g., 2 weeks), Chl levels also decreased dramatically, with <1% of the original Chl remaining (Gorl et al. 1998). Upon addition of nutrients, cyanobacteria regain their blue-green color due to the re-synthesis of PBS complexes. The process of re-synthesis is rapid, and cells can regenerate pigmentation after the readdition of nitrate even after prolonged nitrogen starvation (Gorl et al. 1998).

PBS complex degradation is an active, rapid, and specific process that occurs on a massive scale. Earlier studies monitored the rate of degradation by measuring the decrease of the pigment peaks in whole cell absorption spectra of bulk cultures during nutrient depletion. A model of PBS degradation developed using this approach describes the sequential trimming of the peripheral PC rods, starting at the most distal end, with complete degradation of the remaining PBS occurring within 2 days of continued nutrient depletion in *Synechococcus* sp. PCC 7942 (hereafter referred to as *Synechococcus* 7942) (Collier and Grossman 1992, 1994). To our knowledge, this sequential trimming model of the PBS has not been shown experimentally for *Synechocystis* 6803.

The bulk approach used to examine PBS degradation to date has not allowed for the analysis of individual pigment levels within and between cells [e.g., photosystem I (PSI) compared to photosystem II (PSII) or PC compared to APC] due to their high degree of spectral overlap, nor do the bulk methods provide insight into the changes in the subcellular localization of individual pigments during nutrient starvation. Importantly, single-cell studies performed during nutrient starvation can provide detailed information into the stochastic response of cells within a culture, leading to a direct measure of cell population dynamics. Developments in single-cell, high-content imaging technologies can be applied to answer these questions.

Here, we used hyperspectral confocal fluorescence microscopy (HCFM), a high-content imaging technique, to explore the effect of nitrogen starvation and subsequent PBS degradation on pigment content and localization in live *Synechocystis* 6803 cells. HCFM allows for the spectral resolution of pigments with similar fluorescence emission when combined with multivariate curve resolution (MCR) algorithms, yielding the independently varying fluorescent component spectra that comprise the emission of live cells. As a result, the subcellular distribution of pigments, even those with high spectral overlap, can be shown. HCFM has been previously applied to isolate spectra from highly overlapping pigments in photosynthetic organisms, specifically APC, PC, PSI, and PSII in *Synechocystis* 6803 (Vermaas et al. 2008), and to analyze and compare the distribution of pigments in a group of PBS mutants with increasingly truncated antenna complexes (Collins et al. 2012). The exquisite spectral resolution of HCFM provides multiple advantages for analysis of single-cell behavior in *Synechocystis* 6803—quantification and localization of highly overlapped fluorescence signatures, assessment of cell-to-cell heterogeneity or lack thereof, identification of subpopulations of cells, as well as population dynamics. These advantages are easily extended to other photosynthetic organisms with endogenous pigment fluorescence, as well as exogenously labeled cells in general.

In this study, we explored the plasticity of pigment response in live *Synechocystis* 6803 cells. In a new application of HCFM, we coupled HCFM with single-cell analysis to quantify changes in the spectrally overlapped pigment components in individual wild-type *Synechocystis* 6803 cells during nitrogen starvation to examine the degradation and re-synthesis of PBS on a population level. Our results showed that PBS degradation and re-synthesis are well coordinated, with highly synchronized cell populations undergoing pigment modifications. In addition, Chl fluorescence originating from both PSI and PSII decreased during nitrogen starvation within 24 h, and the phycobilin-to-Chl ratio changed dramatically (~4×) under nitrogen depletion conditions. However, no alteration in subcellular Chl of PBS localization was found. We observed, for the first time, differential rod and core pigment responses to nitrogen deprivation in *Synechocystis* 6803, suggesting that PBS complexes in *Synechocystis* 6803 undergo a stepwise degradation process similar to *Synechococcus* 7942. These data provide insights into how individual pigment-proteins react to changes in extracellular nitrogen at the single-cell and population levels.

## Results

### Bulk pigment response to nitrogen deprivation in Synechocystis 6803 cultures

**Fig. 1:**
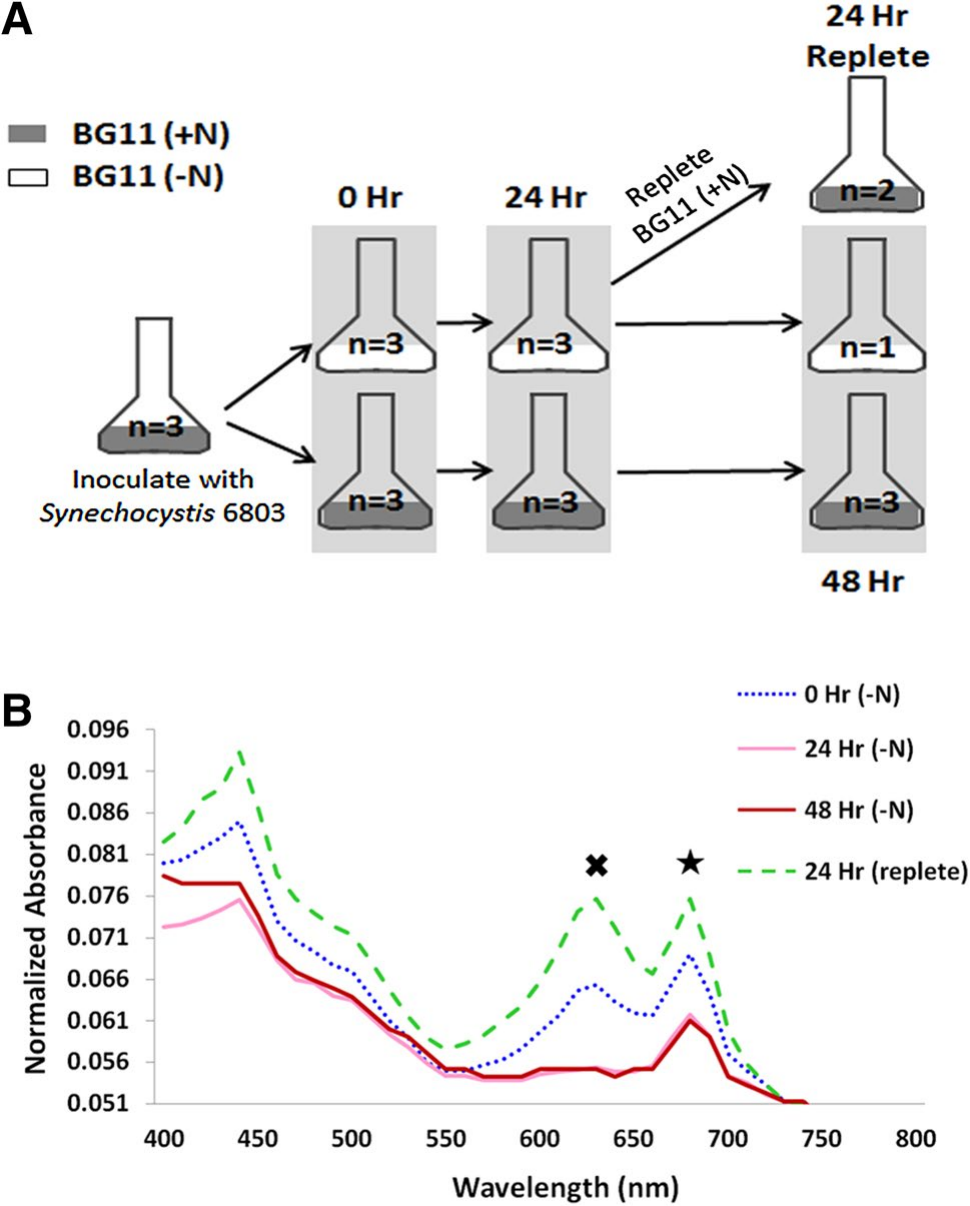
**a** Schematic of experimental design. **b** Bulk absorbance measurements of *Synechocystis* 6803 cultures under varying nitrogen conditions. The average of three biological replicates is represented at 0, 24, and 48 h +N and 0 and 24 h −N. The average of two biological replicates is shown for 24 h post nitrogen repletion. One biological replicate is represented for the 48-h nitrogen-depleted sample. Absorbance spectra are shown normalized to A730. *Cross* indicates the phycobilin peak and *star* indicates the chlorophyll peak

*Synechocystis* 6803 cells were starved of nitrogen and samples were collected at 0-, 24-, and 48-h time points, and at 24 h after nitrogen readdition (Fig. 1a). These samples were used for absorbance measurements and HCFM. The absorbance peak originating from PBS (~625 nm) gradually declined over 24 h of nitrogen depletion and recovered to slightly above original levels upon nitrogen repletion (Fig. 1b). Likewise, the Chl absorbance peak (680 nm) declined with nitrogen depletion and recovered to slightly above original levels when nitrogen was added back to the medium (Fig. 1b).

**Fig. 2:**
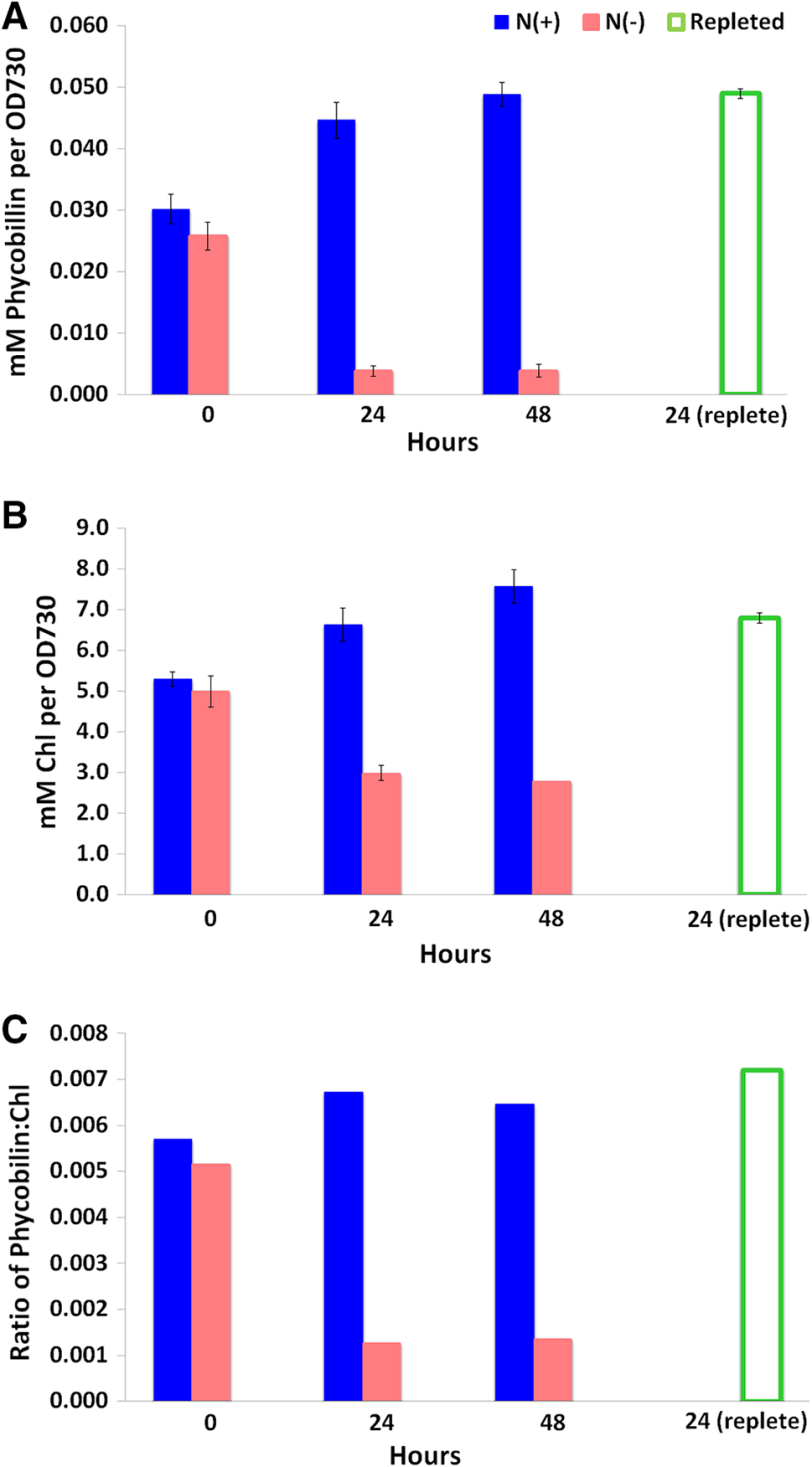
Changes in the concentration of pigments at different time points under varying nitrogen conditions. The average and standard deviation of three biological replicates are represented at 0, 24, and 48 h +N and 0 and 24 h −N. The average and standard deviation of two biological replicates are shown for 24 h post nitrogen repletion. One biological replicate is represented for the 48-h nitrogen-depleted sample. **a** PC content per cell. **b** Chl content per cell. **c** Ratio of phycobilin to chlorophyll content per cell

The pigment abundance per cell can be calculated by dividing by the value at OD_730_, which is an accepted estimate of the number of cells in the culture. The phycobilin and Chl concentrations per OD_730_ were determined from the absorbance measurements at the different time points during nitrogen depletion and repletion (Fig. 2). The phycobilin content OD_730_ in the control cultures (+N) gradually increased by a factor of 1.7 over 48 h, while the phycobilin content OD_730_ in the nitrogen-depleted cultures (−N) dramatically decreased by a factor of 6.5 within 24 h (Fig. 2a). When nitrogen was added back to the depleted culture, the phycobilin concentration per cell increased 12-fold to near control levels. The Chl response to changing nitrogen levels exhibited a pattern similar to phycobilin, though to a lesser extent. The average Chl concentration per OD_730_ decreased by a factor of 1.8 in nitrogen-depleted medium and exhibited a 2.4-fold increase upon nitrogen repletion (Fig. 2b). Interestingly, the average Chl concentration per OD_730_ in the nitrogen-depleted culture did not appear to recover to normal levels 24 h after nitrogen was added back to the medium. The ratio of phycobilin to Chl generally remained constant over 48 h in the cultures with nitrogen in the medium (Fig. 2c). The ratio decreased fivefold in the nitrogen-depleted medium, following the same trend observed in the response of phycobilin to variable nitrogen levels (Fig. 2a). Upon repletion of nitrogen, the ratio of phycobilin to Chl increased sevenfold (Fig. 2c).

### Spectral analysis

**Fig. 3:**
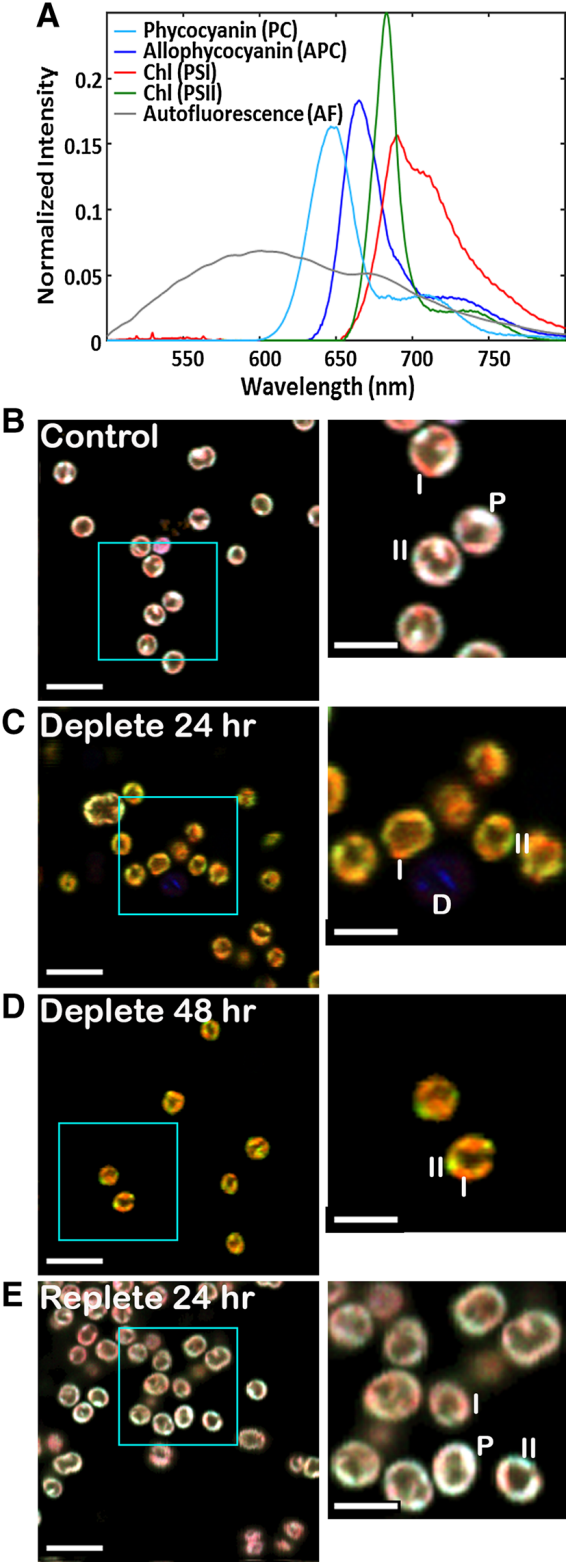
MCR results from hyperspectral confocal fluorescence images of single *Synechocystis* 6803 cells under a time course of varying nitrogen conditions. **a** Spectral model. Fluorescence emission spectra corresponding to four photosynthetic pigments and a broad autofluorescence emission spectrum were mathematically isolated. A sixth spectrum, a flat offset, has been omitted for simplicity. Spectra are normalized to unit length. **b**–**e** RGB images corresponding to the relative abundance of the four photosynthetic pigments. *Red* Chl (PSI), *green* Chl (PSII), *blue* PC + APC. *Scale bars* 5 µm in large images, 2.5 µm in the zoomed inset images. Color scales are identical for all images to facilitate comparison between images. Labels *I, II*, and *P* indicate regions of the cells representative of high concentrations of Chl (PSI), Chl (PSII), and phycobilisomes, respectively. The *D* labels a dead cell consisting primarily of broad autofluorescence

We examined changes in pigments of live, unfixed cells using HCFM. This technique captures an entire emission spectrum from every 3D pixel (voxel) in the image, resulting in data that encompass three spatial dimensions and one spectral dimension. MCR analysis was used to computationally isolate the pure spectral components that contributed to the fluorescence data and to develop a spectral model describing the results (Fig. 3a). Based on this spectral model, independent images that represent the relative abundance and localization of the spectral components assigned to PSII, PSI, PC, and APC are calculated. The images corresponding to the four photosynthetic pigments have been pseudocolored and overlaid for visualization purposes (Fig. 3b–e). The pseudocoloring is based on the colors shown in Fig. 3a: Chl–PSI is red, Chl–PSII is green, and PC and APC are blue. While the color scale has been optimized on a per-component basis to aid in visibility, the color scale is the same for all the images permitting direct comparison of the colors and intensities to assess relative abundance and spatial localization qualitatively.

It should be noted that the HCFM method can also identify dead or dying cells and thus remove their contributions from statistical calculations. The MCR analysis identified a fifth spectral component around 600 nm not directly arising from a photosynthetic pigment (Fig. 3a). This broad cellular autofluorescence peak has been shown to be quite prominent in dead cells and is presumed to arise from the various breakdown products of the photosynthetic pigments including flavonoids, flavins, cinnamic acids, betaxanthine, and pyridine nucleotides (Schulze et al. 2011; Tang and Dobbs 2007). A higher prevalence of dead cells was observed following centrifugation and rinse steps due to the mechanical stress, as expected (data not shown). Additionally, extremely high levels of PBS fluorescence often characterize dying cells. This is because the cells lose the ability to perform energy transfer to reaction centers, resulting in a strong increase in phycobilin fluorescence. Cells with autofluorescence contributions three times the standard deviation of the average population or PC content greater than three times the population average were identified as dead, dying, or severely compromised and excluded from further analysis. On average, this excluded an additional 3–5 cells per condition regardless of time point or nitrogen condition. The ability to include or exclude anomalous cells can improve the validity of the single cell analysis results by providing more accurate average values.

### Single-cell imaging and quantification

Representative, two-dimensional image sections from control (+N) and experimental (–N) cells after 24-h starvation, 48-h starvation, and 24-h repletion are shown in Fig. 3b–e. The (+N) control cells and the 24-h replete cells appeared similar in terms of pigment composition (Fig. 3b, e). The pigments were localized mainly in regions near the cell periphery, consistent with the previously observed location of thylakoid membranes in the cells. Bright punctate regions of heterogeneous fluorescence were observed corresponding to regions where PSI, PSII, and PBS were more concentrated. Examples of these regions are indicated by I, II, and P in the images, respectively.

In contrast, the 24-h (−N) and 48-h (−N) cultures displayed much lower overall fluorescence. In particular, the fluorescence from PC and APC (blue) was greatly diminished after nitrogen starvation, so that the contributions from Chl–PSI (red) and Chl–PSII (green) were more prominent. Bright punctate regions of PSI and PSII localization were still observed at both 24 and 48 h of nitrogen deprivation, indicated by I and II in the images, respectively.

**Fig. 4:**
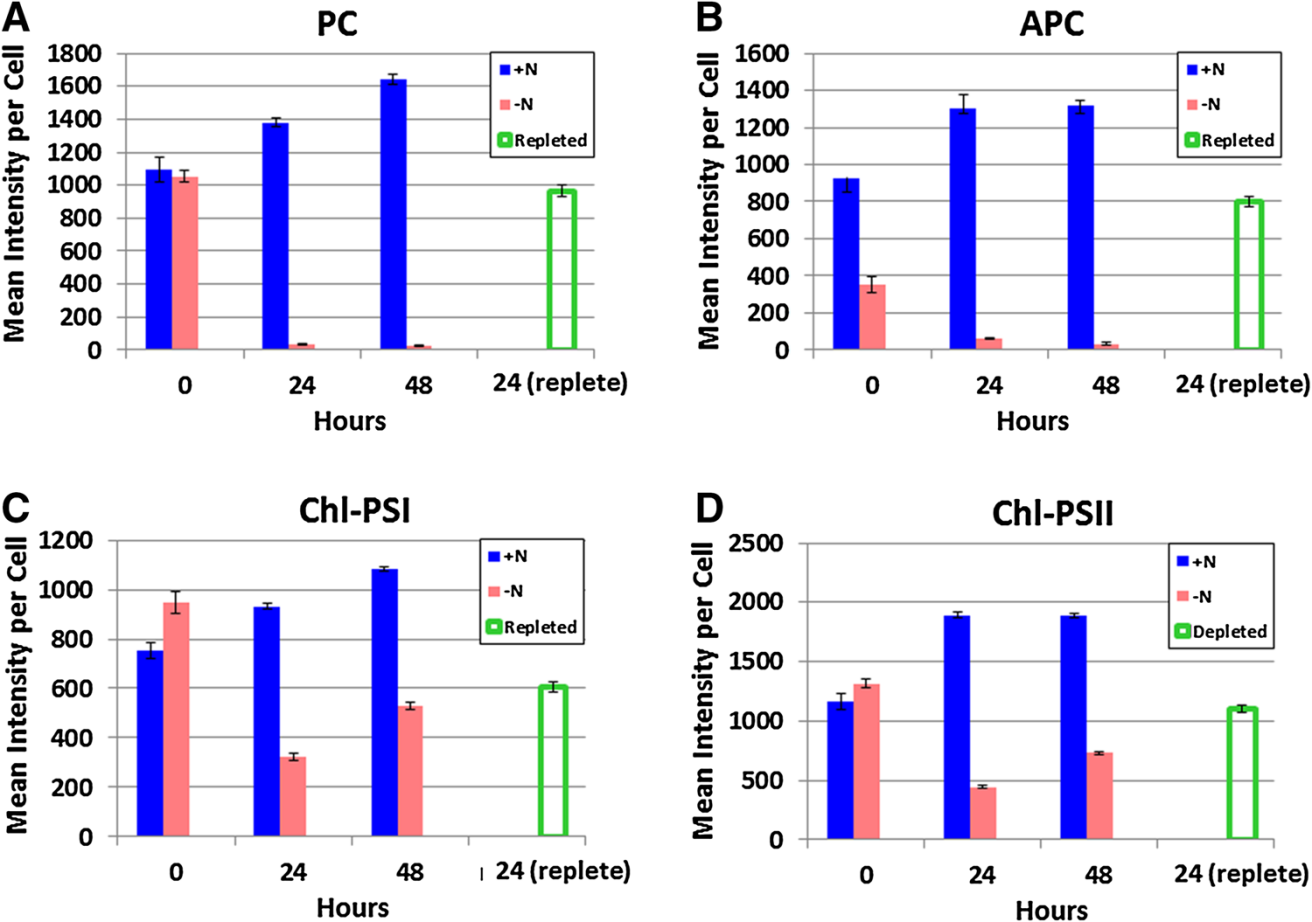
Average per-cell fluorescence intensities of photosynthetic pigments from hyperspectral confocal fluorescence images of *Synechocystis* 6803 under a time course of varying nitrogen conditions. **a** PC, **b** APC, **c** Chl (PSI), and **d** Chl (PSII). *Error bars* represent the standard error of the single-cell measurements

The average per-cell fluorescence intensities of photosynthetic pigments quantified from the HCFM images are shown in Fig. 4. In the nitrogen-starved cells, the intensities of PC (Fig. 4a) and APC (Fig. 4b) were dramatically decreased at 24 and 48 h. The fluorescence intensity for both phycobilin pigments recovered after 24 h of nitrogen readdition, but remained slightly below the levels observed in the 0-h time point. Chl levels for Chl–PSI (Fig. 4c) and Chl–PSII (Fig. 4d) both showed a decline at 24 h followed by a slight increase at 48 h. The replete sample showed a recovery of Chl that remained slightly below the 0-h time point levels. Similiarly to the absorbance measurements, the levels of all pigments in the (+N) control cells showed a slight increase over the course of the experiment from 0 to 48 h. The data in Fig. 4 are summarized in Table 1 as the percent of the initial abundances (*t* = 0).

**Table 1:** Average % of original abundance of photosynthetic pigments following nitrogen depletion (24 h) and repletion (24 h) calculated from the single-cell hyperspectral confocal fluorescence images

Pigment	% of original abundance	
	following N depletion	following N repletion
PC	3 ± 3	88 ± 9
APC	9 ± 1	87 ± 10
Chl–PSI	43 ± 4	80 ± 7
Chl–PSII	38 ± 3	95 ± 8

**Fig. 5:**
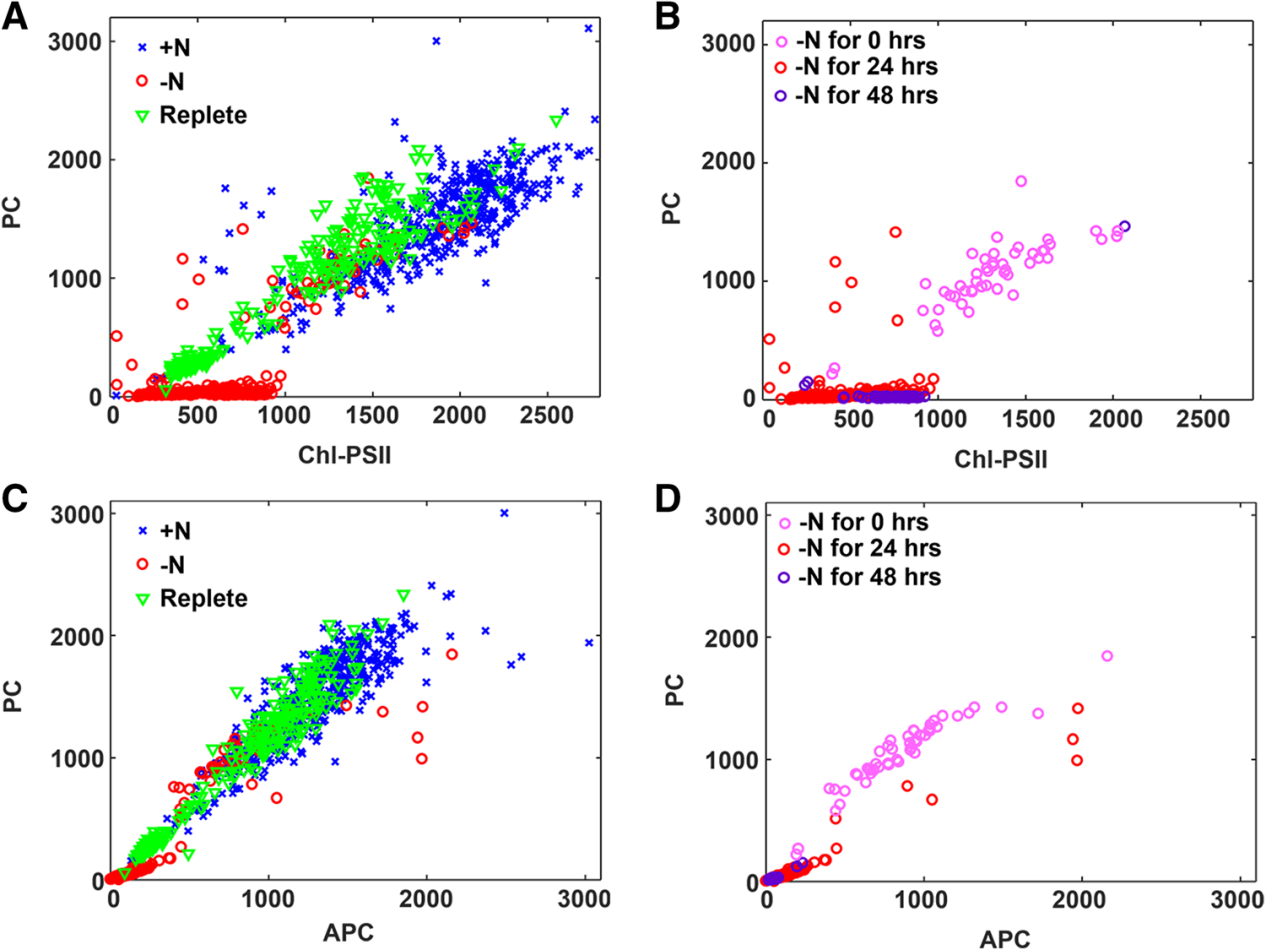
Scatter plots for comparing single-cell abundances of photosynthetic pigments. **a, b** PC versus Chl-PSII content. **c, d** PC versus APC content in individual cells. **a, c** All time points are represented in control (*blue crosses*), nitrogen deplete (*open red circles*), and nitrogen replete conditions (*open green triangles*). **b, d** Data from **a** and **c** showing only deplete conditions and color coding for 0, 24, and 48 h in nitrogen deplete environment

Single-cell scatter plots comparing the pigment abundances in individual cells are shown in Fig. 5. When Chl–PSII was compared to PC (Fig. 5a), the decrease in the levels of both pigments in the nitrogen-starved cells was apparent, especially for PC. When only the nitrogen-depleted cells were examined at the 0-, 24-, and 48-h time points (Fig. 5b), the differences between the time points were seen, as were a few outliers in the 24-h time point that did not show the same pigment changes. When APC versus PC was examined (Fig. 5c), a larger decrease in PC compared to APC was observed upon depletion.

## Discussion

Cyanobacteria harvest light using large PBS antenna systems composed of pigment-proteins. These light-harvesting complexes are degraded during nutrient starvation and are thought to provide a source of nutrients for the cells (Collier and Grossman 1992; Kiyota et al. 2014). While prior research has identified the bulk Chl and PBS abundances during nitrogen starvation in *Synechococcus* 7942 (Collier and Grossman 1992, 1994) and *Synechocystis* 6803 (Baier et al. 2014), subcellular localization and abundance of the individual Chl and PBS pigments remained unknown.

Bulk absorption measurements are the most common method for analyzing pigment content in intact cells. Thus, we compared the single-cell analysis to bulk absorption measurements. We found that the PBS content and Chl levels as measured by bulk absorption increased steadily over 48 h in the control cultures, likely in response to the self-shading occurring during normal cell growth (Shigesada and Okubo 1981). The decreasing PBS and Chl content in response to nitrogen deprivation shown in Fig. 2 are consistent with previous reports (Collier and Grossman 1992) and confirm the experimental conditions for the single-cell measurements. Interestingly, when depletion was rescued by the addition of nitrogen, an increase in the PBS/Chl ratio occurred. This might be due to differences in recovery rates during the 24-h repletion period, with PBS recovery occurring more rapidly. The details of metabolic recovery from nitrogen starvation have begun to be explored in a recent report (Klotz et al. 2016), which outlined the phases of transition from nitrogen starvation to active growth. The second phase of recovery is characterized by repigmentation and resumption of photosynthetic activity.

While these measurements based on absorbance estimate the abundance of Chl and PBS per cell in living cultures, such estimates carry the assumption that all cells have an identical pigment composition. The high degree of spectral overlap among the pigments has prevented direct measurement of the abundance and localization of individual antenna rod (PC) and core (APC) proteins, and PSI with respect to PSII, in single cells. HCFM combined with MCR analysis resolved the overlapping spectra into four pigment components (PSII, PSI, PC, and APC) as shown in Fig. 3a. Figure 3b shows representative images from four conditions measured in this study. The control cells in Fig. 3b show a pigment localization and abundance pattern typical for wild-type *Synechocystis* 6803 cells (Collins et al. 2012; Vermaas et al. 2008). The majority of pigment signal is found in the cell periphery corresponding to the presence of thylakoid membranes, with brighter white foci (500–700 nm in diameter), which suggest that areas of dense thylakoid packing increase the concentration of all pigments (labeled with a ‘P’ in Fig. 3) and the occasional red or green membrane patch, indicating a region of Chl heterogeneity (i.e., either higher or lower Chl–PSI relative to Chl–PSII, labeled with ‘I’ or ‘II’ respectively). Supplementary Figs. 1 and 2 show the spectral data and MCR model for the individual pixels labeled in the insets of Fig. 3b–e. When the cells are depleted of nitrogen, the blue component (PC and APC) simply goes away and the localization of the two Chl components appears unaltered. Both the 24- and 48-h nitrogen-depleted cells clearly show areas of Chl heterogeneity characterized by green (PSII-rich) and red (PSI) patches. Upon repletion, the cells appear indistinguishable from the control cells in terms of pigment localization.

To provide a quantitative assessment of single-cell pigment abundance, the individual cells in the hyperspectral images were segmented and the average abundance of each pigment calculated from individual component images (Fig. 4 and summarized in Table 1). While these results appear similar to the values estimated from the bulk measurements shown in Fig. 2, there are some notable differences that contrast the per-cell pigment abundances estimated by the absorbance measurements (Fig. 2; Table 1). The relative pigment-protein levels per cell in Fig. 2 were calculated using an estimate of the number of cells based on optical density at 730 nm. This is standard practice; however, it should be considered a rough estimate at best because optical density cannot discern whether all cells in the population measured are in the same physiological state. Furthermore, the light scattering properties of cells depend upon cellular ultrastructure, which varies based on the physiological state of the cell (Collier and Grossman 1992).

While Fig. 4 provides quantitation of the spectrally overlapped pigments in individual cells, it is still an average over multiple cells and, thus, does not represent the distribution of cells within the population. The question remains—are all the cells responding in a correlated way or are some cells significantly different from others? Relative pigment abundance was compared at the single-cell level. The data shown in Fig. 5 reveal subtle details about the kinetics of pigment response and cell-to-cell heterogeneity that cannot be uncovered from bulk analysis or even calculating the average pigment abundances in each cell from the high-content hyperspectral images. These points are detailed below.

### Response to nitrogen depletion is coordinated within cell population

From the color groupings in Fig. 5, it is apparent that cells exhibit a high degree of plasticity, with the majority of the cells responding in a concerted way to both nitrogen depletion and repletion. This coordinated response, based on single-cell data, has never been shown experimentally for any pigment, much less highly overlapped pigments like PC, APC, Chl–PSI, and Chl–PSII. Although the majority of the population exhibits a similar physiology, there is evidence of heterogeneity of pigment content in the (+N) control population that has not been previously observed. There are clusters of cells with high PC and low Chl–PSII content as shown in Fig. 5a. This could be indicative of a self-shading response (Shigesada and Okubo 1981). Like Figs. 4, 5b and Supplementary Fig. 3 indicate an increase in the Chl–PSII and Chl–PSI content at 48 h relative to 24-h nitrogen depletion, which is consistent through the entire population of cells (i.e., tight cluster of purple data points). Interestingly, while the majority of the cells respond quickly to (−N) conditions by lowering their PC, APC, Chl–PSII, and Chl–PSI, a subpopulation of cells in (−N) medium at *t* = 24 h respond by raising their PC and APC levels (i.e., red circles apart from the tight red clusters in Fig. 5b, d). These cells may be extremely compromised and no longer capable of performing energy transfer from the PBS to Chl, resulting in an increase in phycobilin fluorescence. By the 48-h time point, these are no longer observed.

### Population heterogeneity is evident in nitrogen replete conditions

Figure 5 also reveals two subpopulations of replete cells (i.e., two separate clusters of green triangles in Fig. 5a, c; Supplementary Fig. 1). While both populations return to the same relative ratio of APC/PC fluorescence (i.e., same slope in Fig. 5c), one has significantly less PC pigments overall and the other has a substantially different Chl–PSII abundance relative to PC. The kinetics of PBS degradation and recovery have been shown to vary within the same population of cells in cyanobacteria (Collier and Grossman 1992). For example, previous studies of nitrogen depletion and repletion in *Synechococcus* 7942 revealed two viability states within the same culture in response to the readdition of nitrogen (Görl et al. 1998). Although population dynamics in response to nutrient availability are known to be heterogeneous in cyanobacteria, the specific divergent cellular processes in these populations have been difficult to resolve. Our results demonstrate a difference in the rate of PC synthesis and PSII activity that is quantified at the single-cell level.

### Initial cell response to nitrogen deplete conditions occurs within minutes

In all the pigment scatter plots, the 0 h nitrogen deplete cells (i.e., cluster of red circles amidst the main blue cluster in Fig. 5a, c and the light pink circles in 5b, d) are centered at slightly lower values than their (+N) counterparts (blue ×’s in Fig. 5a, c). These cells were in the (−N) medium for approximately 5–7 min prior to imaging and the imaging duration was no more than 20 min. These results confirm the rapid cellular response to nitrogen starvation and further quantifies that cells begin to modify their pigment content within 30 min after nitrogen depletion.

### Kinetics of PBS degradation at the single-cell level—the PC rods are degraded prior to the APC core in Synechocystis 6803

It can be seen from Fig. 5c that the ratio of APC/PC fluorescence is about equal in the control (indicated as +N) and replete cells; however, after 24 h of nitrogen depletion (Fig. 5c, d, indicated as −N), the APC/PC ratio increased to approximately 2:1, which is interpreted as a loss of PC relative to APC. This supports the proposed model for sequential PBS degradation in *Synechococcus* 7942 from the periphery of the antenna inward to the central core (Baier et al. 2014; Karradt et al. 2008).

## Experimental procedures

### Cyanobacterial strains and culture conditions

*Synechocystis* 6803 was grown photoautotrophically in BG11 medium with 1.76 M NaNO_3_ as a nitrogen source (Allen 1968). The nitrogen source was replaced with 1.76 M NaCl to generate nitrogen-depleted medium. Cultures were incubated at 30 °C while continuously shaking at 150 rpm (VWR Orbital Shaker) under constant, cool-white light (30 μmol photons m^− 2^ s^− 1^). To perform the experiment, three replicate flasks containing 25 mL BG11 medium were inoculated with *Synechocystis* 6803 (10^7^ cells/mL) and incubated under standard culture conditions. After 3 days, each culture was subdivided into two equal volumes, harvested by gentle centrifugation (500×*g* for 10 min), and washed twice with either nitrogen replete BG11 medium (+N) or nitrogen-depleted medium (−N). After washing, the cells were resuspended in 50 mL BG11 (+N) or BG11 (−N) medium and cultured in a 500-mL Erlenmeyer flask for 24 h. At 24 h, the nitrogen-depleted cultures were harvested by centrifugation as described above and resuspended in either BG11 (+N) or BG11 (−N) medium so that one culture set continues toward chlorosis, while the other set was repleted with nitrogen-enriched medium, respectively. The nitrogen-enriched control cultures were also harvested by centrifugation and resuspended in BG11 (+N) medium to mock the experimental conditions of the test cultures. Samples were obtained for image analysis at 0, 24, and 48 h as shown in Fig. 1a.

### Absorbance analysis

Bulk culture absorbance spectra (400–800 nm) were obtained from all cultures/time points using a plate reader (BioTek Eon). Absorbance spectra were background corrected using the absorbance spectrum from the corresponding cell-free BG-11 (+N or −N) medium. The spectra are shown in Fig. 1b (normalized to 730 nm for visualization and comparison purposes). The absolute concentrations of phycobilin and chlorophyll pigments were calculated using the following equations: 0.139(A620 − A730) − 0.0355(A678 − A730) = mg/mL and 14.96(A678 − A730) − 0.616(A625 − A730) = μg/mL, respectively (Arnon et al. 1974; Collier and Grossman 1992). These values were converted into molarity (mM) by dividing the concentrations obtained with molecular mass of phycobilin (586.67802 g/mol) and chlorophyll (893.48898 g/mol), respectively.

### Single-cell HCFM

Samples for HCFM were prepared by withdrawing 25 µL of the first concentrated cell pellet (prior to any washing steps). This aliquot was resuspended in 100 µL of medium (+N or −N) and 8 µL of the resulting culture was placed on an agar-coated slide. Cells were allowed to settle for 60 s and a coverslip was applied (#1.5), excess culture wicked from the edges, and sealed with nail polish. Imaging was performed immediately.

Hyperspectral confocal fluorescence images were acquired using a custom HCFM described previously (Sinclair et al. 2006). In brief, 3 µW of 488 nm laser excitation was focused onto the sample through a 60x oil immersion objective (Nikon Plan Apochromat; NA = 1.4) to a diffraction-limited spot. Fluorescence emission was collected through the same objective and dispersed by a custom-designed prism spectrometer (Sinclair et al. 2006) onto the focal plane of an electron-multiplied CCD array (iXon DU897U, Andor Technologies). The per-pixel dwell time was 240 ms. The image was formed by raster scanning the beam over the sample with a step size of 0.12 µm. This generates images with diffraction-limited lateral spatial resolution (240 nm). A total of 256 images, each containing 44,100 spectra, were collected.

### Spectral image analysis

Hyperspectral images were preprocessed and subsequently analyzed using multivariate image analysis methods to extract the underlying spectral components and calculate their relative contributions to each image pixel as described (Jones et al. 2012). Representative images from each time point and sample were combined into one image dataset and MCR was executed with non-negativity constraints on all image pixels above the background. This resulted in a six-component spectral model consisting of an instrument offset, PC, APC, Chl in PSII, Chl in PSI, and autofluorescence that explained a total of >99.4% of the spectral variance. The autofluorescent component was included in the analysis to account for dead or dying cells. Concentration maps indicating the abundance and location of each component were generated using a classical least squares analysis with the pure spectra identified from the MCR analysis. The resulting concentration maps were segmented using a modified watershed transformation algorithm to identify individual cells. Automated cell segmentation was verified and edited manually. Single-cell statistics were calculated for individual cells. Dead and dying cells were identified by their extremely high autofluorescent abundance and/or high PBS fluorescence (>3× the average values) and excluded from calculation of single-cell statistics. This resulted in the following number of cells recorded at each condition: T0 + N: 55 cells; T24 + N: 240 cells; T48 + N: 197 cells; T0 –N: 61 cells; T24 –N: 220 cells; T48 –N: 62 cells; T24 replete: 251 cells.

## Electronic supplementary material

Below is the link to the electronic supplementary material.

Supplementary material 1 (TIF 10348 KB)

Supplementary material 2 (TIF 10226 KB)

Supplementary material 3 (TIF 9795 KB)

## References

[CR1] Adir N, Dines M, Klartag M, McGregor A, Melamed-Frank M, Shively JM (2006). Assembly and disassembly of phycobilisomes. Complex intracellular structures in prokaryotes.

[CR2] Allen MM (1968). Simple conditions for growth of unicellular blue-green algae on plates. J Phycol.

[CR3] Arnon DI, McSwain BD, Tsujimoto Y, Wada K (1974). Photochemical activity and components of membrane preparation from blue-green algea: I. Coexistence of two photosystems in relation to chlorophyll a and removal of phycocyanin. Biochem Biophys Acta.

[CR4] Baier K, Nicklisch S, Grundner C, Reinecke J, Lockau W (2001). Expression of two nblA-homologous genes is required for phycobilisome degradation in nitrogen-starved *Synechocystis* sp. PCC6803. FEMS Microbiol Lett.

[CR5] Baier A, Winkler W, Korte T, Lockau W, Karradt A (2014). Degradation of phycobilisomes in *Synechocystis* sp. PCC6803: evidence for essential formation of an NblA1/NblA2 heterodimer and its codegradation by a Clp protease complex. J Biol Chem.

[CR6] Bogorad L (1975). Phycobiliproteins and complementary chromatic adaptation. Annu Rev Plant Physiol.

[CR7] Buick R (2008). When did oxygenic photosynthesis evolve?. Philos Trans R Soc B.

[CR8] Collier JL, Grossman AR (1992). Chlorosis induced by nutrient deprivation in *Synechococcus* sp. strain PCC 7942: not all bleaching is the same. J Bacteriol.

[CR9] Collier JL, Grossman AR (1994). A small polypeptide triggers complete degradation of light-harvesting phycobiliproteins in nutrient-deprived cyanobacteria. EMBO J.

[CR10] Collins AM, Liberton M, Garcia OF, Jones HDT, Pakrasi HB, Timlin JA (2012). Photosynthetic pigment localization and thylakoid membrane morphology are altered in *Synechocystis* 6803 phycobilisome mutants. Plant Physiol.

[CR11] Dolganov N, Grossman AR (1999). A polypeptide with similarity to phycocyanin α-subunit phycocyanobilin lyase involved in degradation of phycobilisomes. J Bacteriol.

[CR12] Gorl M, Sauer J, Baier T, Forchhammer K (1998). Nitrogen-starvation-induced chlorosis in *Synechococcus* PCC 7942: adaptation to long-term survival. Microbiology.

[CR13] Görl M, Sauer J, Baier T, Forchhammer K (1998). Nitrogen-starvation-induced chlorosis in *Synechococcus* PCC 7942: adaptation to long-term survival. Microbiology.

[CR14] Grossman AR, Schaefer MR, Chiang GG, Collier JL (1993). The phycobilisome, a light-harvesting complex responsive to environmental conditions. Microbiol Mol Biol Rev.

[CR15] Grossman AR, Bhaya D, He Q (2001). Tracking the light environment by cyanobacteria and the dynamic nature of light harvesting. J Biol Chem.

[CR16] Jones HDT, Haaland DM, Sinclair MB, Melgaard DK, Collins AM, Timlin JA (2012). Preprocessing strategies to improve MCR analyses of hyperspectral images. J Chemom Intell Lab Syst.

[CR17] Karradt A, Sobanski J, Mattow J, Lockau W, Baier K (2008). NblA, a key protein of phycobilisome degradation, interacts with ClpC, a HSP100 chaperone partner of a cyanobacterial Clp protease. J Biol Chem.

[CR18] Kiyota H, Hirai MY, Ikeuchi M (2014) NblA1/A2-dependent homeostasis of amino acid pools during nitrogen starvation in Synechocystis sp. PCC 6803. Metabolites 4(3):517–531. doi:10.3390/metabo4030517 10.3390/metabo4030517 PMC4192677 24983765

[CR19] Klotz A (2016). Awakening of a dormant cyanobacterium from nitrogen chlorosis reveals a genetically determined program. Curr Biol.

[CR20] Li H, Sherman LA (2002). Characterization of *Synechocystis* sp. strain PCC 6803 and ∆nbl mutants under nitrogen-deficient conditions. Arch Microbiol.

[CR21] MacColl R (1998). Cyanobacterial phycobilisomes. J Struct Biol.

[CR22] Nguyen AY, Bricker WP, Zhang H, Weisz DA, Gross ML, Pakrasi HB (2017). The proteolysis adaptor, NblA, binds to the N-terminus of β-phycocyanin: implications for the mechanism of phycobilisome degradation. Photosynth Res.

[CR23] Schulze K, López DA, Tillich UM, Frohme M (2011). A simple viability analysis for unicellular cyanobacteria using a new autofluorescence assay, automated microscopy, and ImageJ. BMC Biotechnol.

[CR24] Schwarz R, Forchhammer K (2005). Acclimation of unicellular cyanobacteria to macronutrient deficiency: emergence of a complex network of cellular responses. Microbiology.

[CR25] Shigesada N, Okubo A (1981). Analysis of the self-shading effect on algal vertical distribution in natural waters. J Math Biol.

[CR26] Sinclair MB, Haaland DM, Timlin JA, Jones HDT (2006). Hyperspectral confocal microscope. Appl Opt.

[CR27] Tang YZ, Dobbs FC (2007). Green autofluorescence in dinoflagellates, diatoms, and other microalgae and its implications for vital staining and morphological studies. Appl Environ Microbiol.

[CR28] van Waasbergen LG, Dolganov N, Grossman AR (2002). nblS, a gene involved in controlling photosynthesis-related gene expression during high light and nutrient stress in *Synechococcus elongatus* PCC 7942. J Bacteriol.

[CR29] Vermaas WF, Timlin JA, Jones HD, Sinclair MB, Neiman LT, Hamad SW, Melgaard DK, Haaland DM (2008). In vivo hyperspectral confocal fluorescence imaging to determine pigment localization and distribution in cyanobacterial cells. PNAS.

[CR30] Watanabe M, Ikeuchi M (2013). Phycobilisome: architecture of a light-harvesting supercomplex. Photosynth Res.

[CR31] Wegener KM, Singh AK, Jacobs JM, Elvitigala T, Welsh EA, Keren N, Gritsenko MA, Ghosh BK, Cambll DG, Smith RD, Pakrasi HB (2010). Global proteomics reveal an atypical strategy for carbon/nitrogen assimilation by a Cyanobacterium under diverse environmental perturbations. Mol Cell Proteom.

