# Supplementary figures and images for: Population-level coordination of pigment response in individual cyanobacterial cells under altered nitrogen levels
Source: Photosynth Res. 2017 Jul 21;134(2):165–74. doi: 10.1007/s11120-017-0422-7 (PMC5645440; doi:10.1007/s11120-017-0422-7)

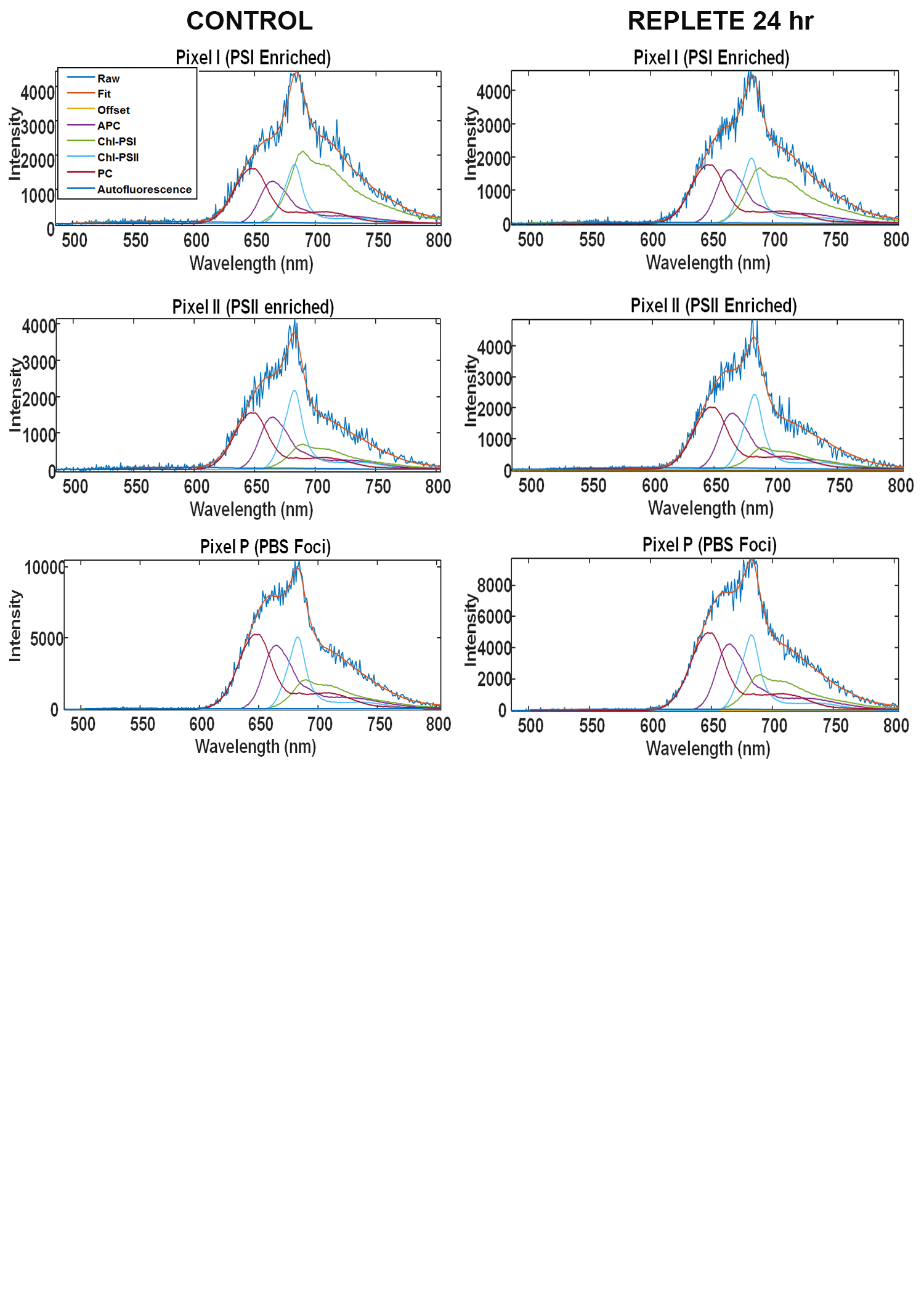

Supplement: Supplementary file 1 — Supplementary material 1 (TIF 10348 KB) [file 11120_2017_422_MOESM1_ESM.tif]

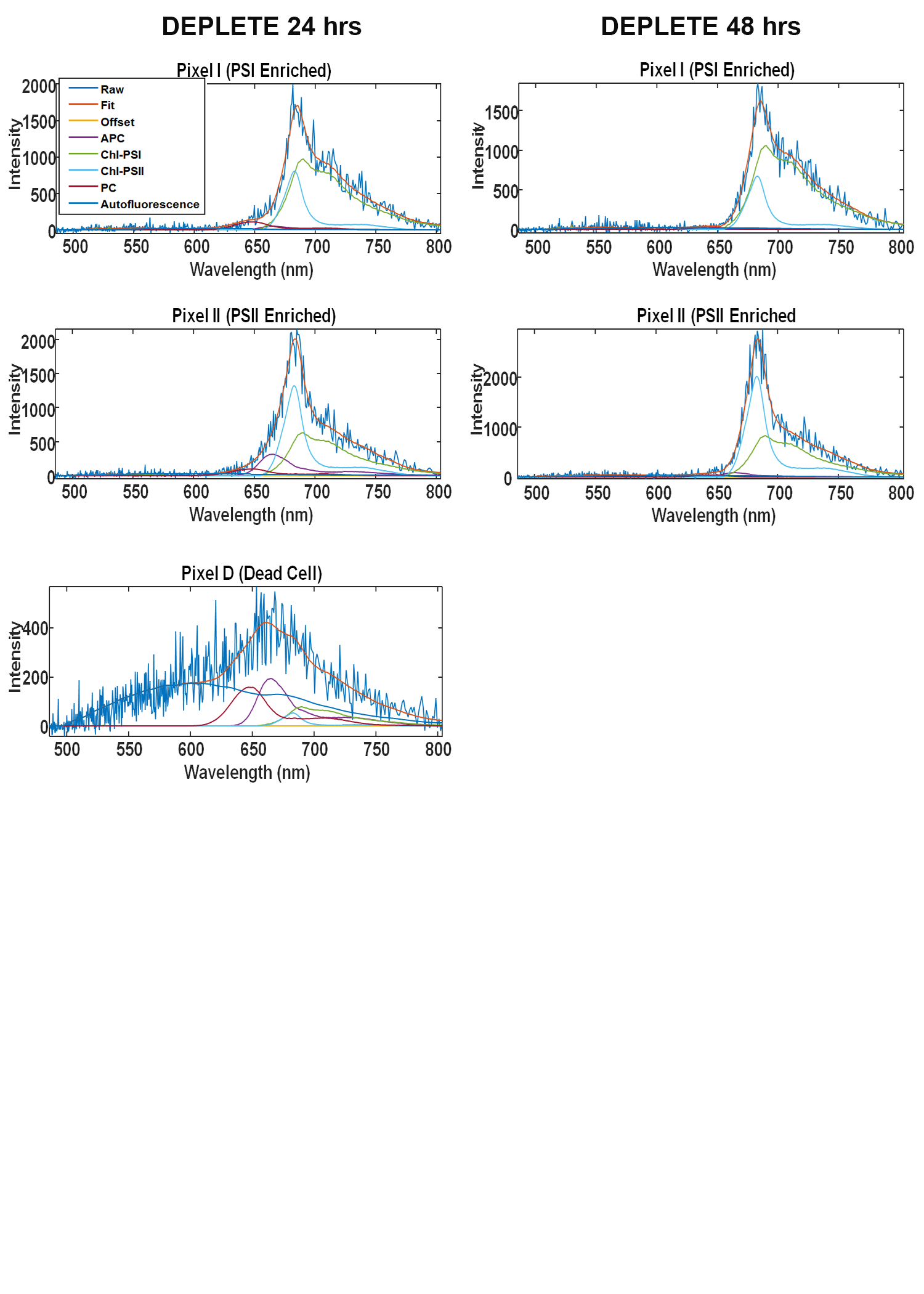

Supplement: Supplementary file 2 — Supplementary material 2 (TIF 10226 KB) [file 11120_2017_422_MOESM2_ESM.tif]

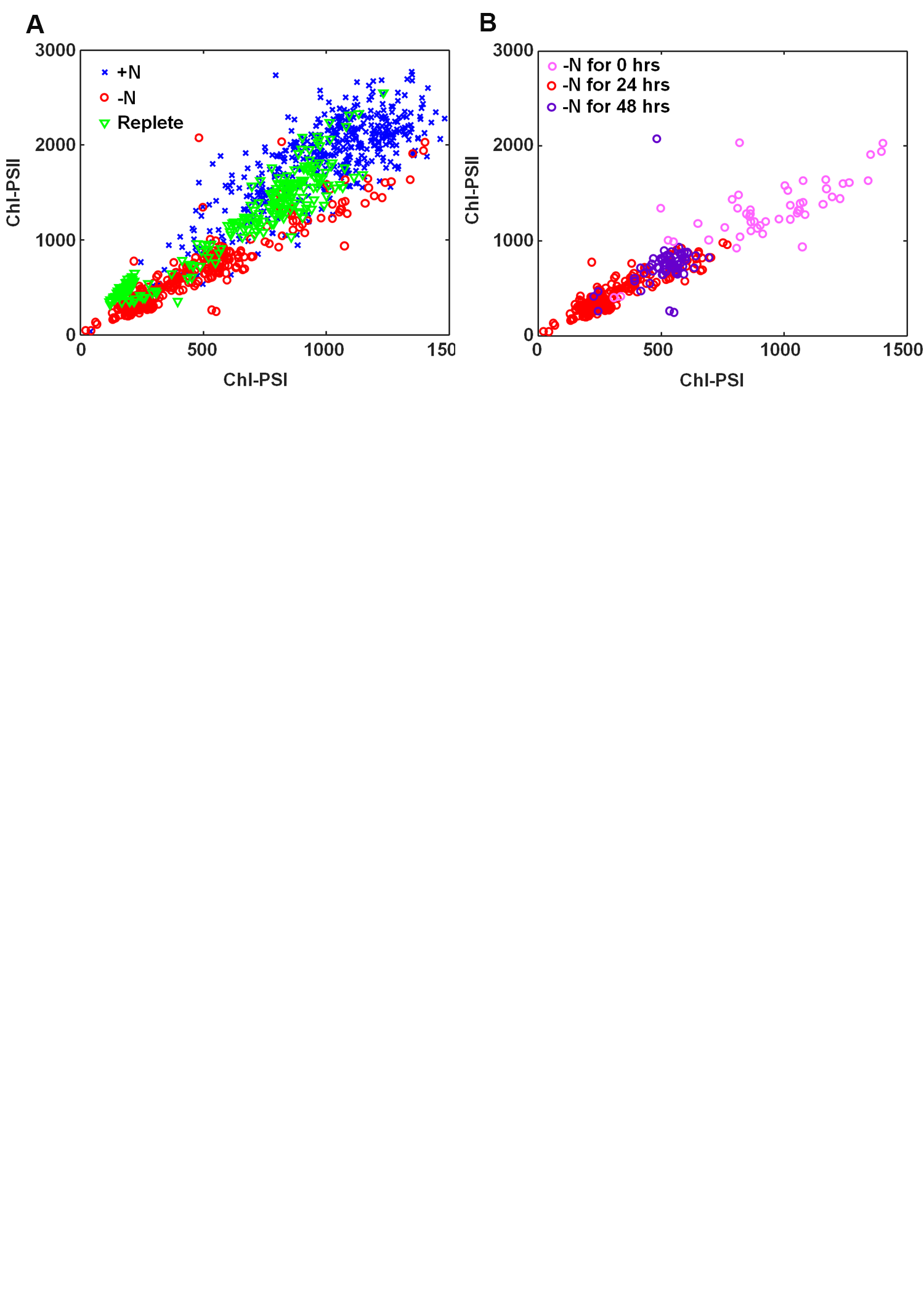

Supplement: Supplementary file 3 — Supplementary material 3 (TIF 9795 KB) [file 11120_2017_422_MOESM3_ESM.tif]
